# Derivatives of the Antimicrobial Peptide BP100 for Expression in Plant Systems

**DOI:** 10.1371/journal.pone.0085515

**Published:** 2013-12-23

**Authors:** Esther Badosa, Gemma Moiset, Laura Montesinos, Montserrat Talleda, Eduard Bardají, Lidia Feliu, Marta Planas, Emilio Montesinos

**Affiliations:** 1 Laboratory of Plant Pathology, Institute of Food and Agricultural Technology-CIDSAV-XaRTA, University of Girona, Campus Montilivi, Girona, Spain; 2 LIPPSO, Department of Chemistry, University of Girona, Campus Montilivi, Girona, Spain; National Taiwan University, Taiwan

## Abstract

Production of antimicrobial peptides in plants constitutes an approach for obtaining them in high amounts. However, their heterologous expression in a practical and efficient manner demands some structural requirements such as a minimum size, the incorporation of retention signals to assure their accumulation in specific tissues, and the presence of protease cleavage amino acids and of target sequences to facilitate peptide detection. Since any sequence modification may influence the biological activity, peptides that will be obtained from the expression must be screened prior to the synthesis of the genes for plant transformation. We report herein a strategy for the modification of the antimicrobial undecapeptide **BP100** that allowed the identification of analogues that can be expressed in plants and exhibit optimum biological properties. We prepared 40 analogues obtained by incorporating repeated units of the antimicrobial undecapeptide, fragments of natural peptides, one or two AGPA hinges, a Gly or Ser residue at the N-terminus, and a KDEL fragment and/or the epitope tag54 at the C-terminus. Their antimicrobial, hemolytic and phytotoxic activities, and protease susceptibility were evaluated. Best sequences contained a magainin fragment linked to the antimicrobial undecapeptide through an AGPA hinge. Moreover, since the presence of a KDEL unit or of tag54 did not influence significantly the biological activity, these moieties can be introduced when designing compounds to be retained in the endoplasmic reticulum and detected using a complementary epitope. These findings may contribute to the design of peptides to be expressed in plants.

## Introduction

 Antimicrobial peptides (AMPs) are short sequences containing less than 50 amino acids. They are considered a first line of defense in plants and animals or are produced by microorganisms participating in antibiosis processes [1]. There is broad literature review on AMPs produced in bacteria [2-4], fungi [5,6], insects [7,8], marine invertebrates [9], amphibian, mammals [10,11], and plants [12].

Due to their potential high biocompatibility, moderate biodegradability, and low resistance developed on target microorganisms, AMPs offer great perspectives as a novel class of antibiotics with application in several fields. They can be used to combat fungal and bacterial infections in humans [7,10] and plant diseases in crop protection [1,13,14]. Moreover, they can substitute or complement antibiotics in animal feed, biopreservatives in food, cosmetics and biomaterials, and antifoulings [15,16]. However, the exploitation of AMPs encounters several difficulties because they are produced at low concentrations in living organisms and often their antimicrobial activity is low to moderate. In addition, some of the AMPs showing high antimicrobial activity may be relatively toxic to non-target organisms (animals, humans, plants).

To overcome the above limitations, novel peptides have been designed based on structure-activity relationship studies in natural AMPs. Small truncated sequences containing the minimal domain for activity have been developed as well as chimeric constructions. De novo designed sequences, bearing structural features that are crucial for the activity of natural peptides, have also been reported. Combinatorial chemistry approaches are also powerful tools that have been used to optimize the biological activity profile of AMPs, and sequences with improved activity, decreased toxicity to non-target organisms and low susceptibility to proteolytic hydrolysis have been identified. Following this rationale, we have designed chimeric peptides that are cecropin A-melittin hybrids and their biological activity has been optimized through the synthesis of a 125-member library (CECMEL11) [17]. From this library we have identified **BP100** and several analogues active against bacterial and fungal phytopathogens with minimal inhibitory concentrations (MIC) lower than 10 μM [17-19]. This activity is relevant because it is of the same order than that of standard antibiotics and antifungals (e.g. penicillins, aminoglycosides, ketoconazole). Moreover, they showed an extremely high biocompatibility with an acute oral toxicity, determined as the LOD_50_, higher than 2000 mg/Kg of body weight in mice [20]. 

**BP100** and its derivatives have strong cationic charge and amphipathic arrangement that enable their interaction with biological membranes resulting in cell membrane disruption. Biophysical studies with **BP100** using phospholipid bilayers similar to that of the bacterial cytoplasmic membrane showed vesicle permeabilization, membrane electroneutrality, and vesicle aggregation, but also translocation [21]. It has been also reported that **BP100** is a fast and efficient cell-penetrating agent to deliver functional cargoes peptides into tobacco cells [22].

Exploitation of AMPs may be performed by expression in plants for self-defence against bacterial or fungal pathogens or through mass production to be used as active ingredients in antimicrobial formulations. Mass production can be accomplished through chemical or quimioenzymatic synthesis, or by means of microbial or plant biofactories. Chemical synthesis using solution or solid-phase protocols or employing enzymatic procedures is only economically feasible for the preparation of short peptides. In contrast, heterologous production of AMPs using living systems as biofactories offers a reliable and sustainable mean of exploitation of these peptides which can lead to high amounts of product. Microbial systems, such as *Escherichia coli* or *Pichia pastoris*, have been reported as efficient production platforms for several proteins [23,24]. Plants have also been used as biofactories for the production of medium to large size proteins due to their high added value for pharmaceutical applications [25-28]. 

Linear AMPs containing proteinogenic aminoacids can also be produced in plants [13,29]. However, to be heterologous expressed in plants, peptides must fulfill some structural requirements. Particularly, the size of the peptide has to be above a minimum expressability threshold [30]. Moreover, the peptide has to be targeted to subcellular organelles to guarantee its stability and avoid cell toxicity [31]. In addition, the expression and accumulation of the peptide in specific plant tissues is necessary to meet low cost production needs [32] and to decrease downstream processing operations [33,34]. To accomplish these requirements the following strategies may be used: (i) increase of the peptide length by n-merizations, chimeric enlargements or fusions [35], (ii) stabilization/distortion by incorporating an AGPA hinge between the peptide fragments [36], (iii) introduction of retention signals for the accumulation of peptides in subcellular organelles, such as the endoplasmic reticulum by the addition of the KDEL sequence at the C-terminus [13,37], (iv) incorporation of protease cleavage amino acids for processing fusions [38], and (v) of target sequences to allow peptide detection and/or purification [39,40].

Taking into account these considerations, to achieve an efficient production of the CECMEL11 peptides in living systems and, particularly, in plants, the sequences to be expressed should incorporate the above structural features. Unfortunately, any sequence modification may have dramatical consequences in the peptide properties, including antimicrobial, hemolytic and phytotoxic activities as well as protease susceptibility, as it has been previously described [17,19,20]. Therefore, the biological activity of the sequences that will be obtained from the expression process must be screened prior to the synthesis of the corresponding genes and the cloning systems for plant transformation. 

In the present work, we designed, synthesized and evaluated 40 sequences derived from **BP100** for their antimicrobial, hemolytic and phytotoxic activities, and protease susceptibility. These analogues were designed based on the structural requirements that they must possess to be expressed in plants. Finally, the best candidates are proposed for further development of transgenic plants for their production.

## Materials and Methods

### Peptide synthesis

 All peptides were synthesized manually by the solid-phase method using 9-fluorenylmethoxycarbonyl (Fmoc)-type chemistry, tert-butyloxycarbonyl side-chain protection for Lys and Trp, tert-butyl (*t*Bu) for Tyr, Glu, Asp, Thr, Gln and Ser, and trityl for His. An aminomethyl ChemMatrix resin (0.59 mmol/g) was used as solid support. The linker 3-(4-hydroxymethylphenoxy)propionic acid (PAC) was employed to obtain C-terminal peptide acids. The PAC linker (3 equiv) was coupled to the resin with *N*-[1*H*-benzotriazol-1-yl)(dimethylamino)methylene]-*N*-methylmethanaminium hexafluorophosphate *N*-oxide (HBTU) (3 equiv) and *N,N*-diisopropylethylamine (DIEA) (3 equiv) in *N,N*-dimethylformamide (DMF) for 5 h, and the reaction was monitored by the ninhydrin test. The coupling of the first amino acid (5 equiv) was performed using *N,N*-diisopropylcarbodiimide (DIPCDI) (5 equiv), 1-hydroxybenzotriazole (HOBt) (5 equiv) and *N,N*-dimethylaminopyridine (DMAP) (1.25 equiv) in DMF for 15 h. After the coupling, an Fmoc test was performed to check the resin loading. The resin was then acylated by treatment with a mixture of acetic anhydride−pyridine−CH_2_Cl_2_ (1:1:1; 2 × 30 min). Couplings of the other Fmoc-amino acids (4 equiv) were mediated by HBTU (3.8 equiv), HOBt (4 equiv) and DIEA (7.8 equiv) in DMF for 1 h, and monitored by the ninhydrin test. Fmoc group was removed by treating the resin with a mixture of piperidine−DMF (3:7; 2 + 10 min). Peptides were individually cleaved from the resin with trifluoroacetic acid (TFA)-H_2_O−triisopropylsilane (95:2.5:2.5; 2 h). Following TFA evaporation and diethyl ether extraction, the crude peptides were dissolved in H_2_O and lyophilized.

Peptides were analyzed under standard analytical high-performance liquid chromatography (HPLC) conditions with a Dionex liquid chromatography instrument (Conditions A-D). Detection was performed at 220 nm. Solvent A was 0.1% aqueous TFA and solvent B was 0.1% TFA in CH_3_CN. Conditions A: Analysis was carried out with a Kromasil 100 C_18_ (4.6 mm × 40 mm, 3.5 μm) column with a 2–100% B over 7 min at a flow rate of 1 ml/min. Conditions B: Analysis was carried out with a Kromasil 100 C_18_ (4.6 mm × 40 mm, 3.5 μm) column with a 30–50% B over 6 min at a flow rate of 1 ml/min. Conditions C: Analysis was carried out with a Kromasil 100 C_18_ (4.6 mm × 40 mm, 3.5 μm) column with a 60–70% B over 6 min at a flow rate of 1 ml/min. Conditions D: Analysis was carried out with a Kromasil 100 C_18_ (4.6 mm × 250 mm, 3.5 μm) column with a 2–100% B over 30 min at a flow rate of 1 ml/min. Other peptides were analyzed under standard analytical HPLC conditions with an Agilent Technologies 1200 Series liquid chromatography instrument (Conditions E). Detection was performed at 220 nm. Solvent A was 0.1% aqueous TFA and solvent B was 0.1% TFA in CH_3_CN. Conditions E: Analysis was carried out with a Kromasil 100 C_18_ (4.6 mm × 40 mm, 3.5 μm) column with a 2–100% B over 5 min at a flow rate of 1 ml/min.

Electrospray ionization mass spectrometry (ESI-MS, Bruker Daltonics, USA) and matrix-assisted laser desorption ionization with time-of-flight analysis (MALDI-TOF, Bruker, USA) were used to confirm peptide identity. 

Peptides **BP216**, **BP217**, **BP235** and **BP236** were purchased from CASLO Laboratory ApS (Lyngby, Denmark) at >90% purity.

### Bacterial strains and growth conditions

 For the analysis of the in vitro activity of peptides, the following plant pathogenic bacterial strains were used: *E. amylovora* PMV6076 (Institut National de la Recherche Agronomique, Angers, France), *P. syringae* pv. syringae EPS94 (Institut de Tecnologia Agroalimentària, Universitat de Girona, Spain) and *X. axonopodis* pv. vesicatoria 2133-2 (Instituto Valenciano de Investigaciones Agrarias, Valencia, Spain). All bacteria were stored in Luria Bertani (LB) broth supplemented with glycerol (20%) and maintained at -80 °C. All strains were scrapped from LB agar plates incubated at 25 °C after growing for 24 h in the case of *E. amylovora* and *P. syringae* pv. syringae, and for 48 h for *X. axonopodis* pv. vesicatoria. The cell material was suspended in sterile water to obtain a suspension of 10^8^ CFU ml^-1^. 

### Antibacterial activity

 Lyophilized peptides were solubilized in sterile distilled water to a concentration of 1000 μM and filter sterilized through a 0.22-μm-pore-size filter. For MIC assessment, dilutions of the synthetic peptides were made to obtain a final concentration of 200, 100, 75, 50, 25 and 12.5 μM. Twenty microlitres of each dilution were mixed in a microtiter plate well with 20 μl of the corresponding suspension of the bacterial indicator at 10^8^ CFU ml^-1^ and with 160 μl of Trypticase Soy Broth (TSB) (BioMèrieux, France) to a total volume of 200 μl. Final peptide concentrations assayed were 20, 10, 7.5, 5, 2.5 and 1.25 μM. **Tag54** and **tag54-2** were tested at 100 μM. Three replicates for each strain, peptide and concentration were used. Positive controls contained water instead of peptide and negative controls contained peptide without bacterial suspension. Microbial growth was automatically determined by optical density measurement at 600 nm (Bioscreen C, Labsystem, Finland). Microplates were incubated at 25 °C with 20 s shaking before hourly absorbance measurement for 48 h. The experiment was repeated twice. The MIC was taken as the lowest peptide concentration with no growth at the end of the experiment.

### Hemolytic activity

 The hemolytic activity of peptides was evaluated by determining hemoglobin release from erythrocyte suspensions of fresh human blood (5%, vol/vol). Blood was aseptically collected using a BD vacutainer K2E System with EDTA (Belliver Industrial State, Plymouth, U.K.) and stored for less than 2 h at 4 °C. Blood was centrifuged at 6,000 × g for 5 min, washed three times with TRIS buffer (10 mM TRIS, 150 mM NaCl, pH 7.2) and ten-fold diluted in the same buffer. Peptides were solubilized in TRIS buffer to a concentration of 500, 300 and 100 μM. Sixty five microliters of human red blood cells were mixed with 65 μl of the peptide solution (final concentration of 250, 150 and 50 μM) in a 96-well reaction plate and incubated under continuous shaking for 1 h at 37 °C. Then, the plates were centrifuged at 3,500 × g for 10 min. Eighty microliter aliquots of the supernatant were transferred to 100-well microplates and diluted with 80 μl of sterile distilled water. Three replicates for each peptide were used. Hemolysis was measured as the absorbance at 540 nm with a microplate reader. Complete hemolysis was determined in TRIS buffer plus melittin at 100 μM as a positive control. The percentage of hemolysis (*H*) was calculated using the equation: *H* = 100 × [(*Op* − *Ob*)/(*Om* − *Ob*)], where *Op* is the density for a given peptide concentration, *Ob* for the buffer, and *Om* for the melittin positive control.

### Phytotoxicity

A set of 16 selected peptides **BP100**, **BP134**, **BP173**, **BP178**, **BP183**, **BP188**, **BP192**, **BP209**, **BP210**, **BP211**, **BP213**, **BP214**, **BP215**, **BP216**, **BP217** and **BP235** were evaluated for their phytotoxicity. Tobacco plants (*Nicotiana benthamiana*) were grown from seed in the glasshouse and used between 20 and 30 days old. One hundred μl of the peptides at concentrations from 50 to 250 μM, depending on the peptide, were inoculated into the mesophylls of tobacco leaves as described previously [41], and plants were incubated again for three days. Up to six independent inoculations were carried out in a single leaf, and at least three independent inoculations were performed per peptide and concentration, randomly distributed in different leaves and plants. Toxicity was measured as the lesion diameter.

### Ethics statement

The use of human blood samples was solely to assess the hemolytic activity of the peptides, and was not used for other type of research with ethics concerns. The president of the Research Committee of the University of Girona confirmed that ethics approval was not required for the collection of blood samples.

## Results

### Design and synthesis of peptides

 Peptides were designed and synthesized in order to obtain sequences with optimized properties for plant expression at high yield and for accumulation in different subcellular plant cell compartments. Peptide sequences were based on the antimicrobial peptide **BP134** (KKLFKKILKYL-OH), a C-terminal carboxylic acid derivative of **BP100** [17]. A total of 40 peptides of 15 to 52 amino acids in length were prepared. One set of peptides contained one, two or three units of **BP134** (Table 1) and a second set incorporated a combination of one unit of **BP134** with: (i) melittin(10-19) or melittin(1-13); (ii) magainin(4-10); (iii) magainin(1-10); and (iv) cecropin A(25-37) (Table 2). Analogues of these peptides were also obtained by introducing: (i) one or two AGPA hinges as stabilization/distortion moiety; (ii) a KDEL fragment at the C-terminus as a signal for permanent retention of peptides in the endoplasmic reticulum; (iii) a Gly or a Ser residue at the N-terminus as a TEV protease recognition site; and (iv) the epitope tag KDWEHLKDWEHLKDWEHL (**tag54**) at the C-terminus for peptide detection or purification.

**Table 1:** Sequences, number of amino acids, retention times and purities on HPLC, and mass spectrometry data of peptides from the first set.

Peptide	Sequence	#Aa	*t*_R_ (min)^a^	Purity^b^ (%)	Theoretical monoisotopic *m/z*	Observed *m/z* ^c^
*BP134 monomers*						
**BP134**	KKLFKKILKYL-OH	11	6.24^d^	90	1421.9	1422.1 [M+H]^*+*^
**BP199**	KKLFKKILKYL-AGPA-OH	15	6.60^d^	89	1718.1	1718.3 [M+H]^*+*^
**BP214**	KKLFKKILKYL-KDEL-OH	15	3.14^e^	88	1907.2	1907.2 [M+H]^*+*^
*BP134 dimers*						
**BP203**	KKLFKKILKYL-KKLFKKILKYL-OH	22	7.48^d^	85	2824.9	2824.9 [M+H]^*+*^
**BP202**	KKLFKKILKYL-AGPA-KKLFKKILKYL-OH	26	3.58^e^	99	3121.1	3121.3 [M+H]^*+*^
**BP193**	G-KKLFKKILKYL-AGPA-KKLFKKILKYL-OH	27	7.18^d^	89	3179.1	3178.8 [M+H]^*+*^
**BP195**	S-KKLFKKILKYL-AGPA-KKLFKKILKYL-OH	27	7.19^e^	93	3209.1	3208.7 [M+H]^*+*^
**BP200**	KKLFKKILKYL-AGPA-KKLFKKILKYL-AGPA-OH	30	7.53^e^	68	3417.2	3417.4 [M+H]^*+*^
**BP198**	KKLFKKILKYL-KKLFKKILKYL-KDEL-OH	26	4.08^e^	99	3311.3	3311.5 [M+H]^*+*^
**BP192**	KKLFKKILKYL-AGPA-KKLFKKILKYL-KDEL-OH	30	7.27^d^	95	3607.5	3608.0 [M+H]^*+*^
**BP213**	KKLFKKILKYL-AGPA-LYKLIKKFLKK-KDEL-OH	30	3.48^e^	90	3606.3	3606.3 [M+H]^*+*^
**BP194**	G-KKLFKKILKYL-AGPA-KKLFKKILKYL-KDEL-OH	31	7.33^d^	91	3664.6	3665.0 [M+H]^*+*^
**BP196**	S-KKLFKKILKYL-AGPA-KKLFKKILKYL-KDEL-OH	31	7.35^d^	71	3694.6	3695.0 [M+H]^*+*^
**BP236**	KKLFKKILKYL-AGPA-KKLFKKILKYL-AGPA-KDWEHLKDWEHLKDWEHL-KDEL-OH	52	20.30^*f*^	99	6330.7 [M+H]^*+*^	1266.9 [M+5H]^5+^
*BP134 trimers*						
**BP204**	KKLFKKILKYL-KKLFKKILKYL-KKLFKKILKYL-OH	33	9.27^d^	52	4228.8	4229.3 [M+H]^*+*^
**BP201**	KKLFKKILKYL-AGPA-KKLFKKILKYL-AGPA-KKLFKKILKYL-OH	41	8.13^d^	67	4821.1	4821.4 [M+H]^*+*^
**BP216**	KKLFKKILKYL-AGPA-KKLFKKILKYL-AGPA-KKLFKKILKYL-KDEL-OH	45	4.18^e^	99	5307.8 [M+H]^*+*^	885.3 [M+6H]^6+^
*Tags*						
**tag54**	KDWEHLKDWEHLKDWEHL-OH	18	2.95^e^	99	2443.7	2443.2 [M+H]^*+*^
**tag54‑2**	KDWEHLKDWEHLKDWEHL-KDEL-OH	22	2.96^e^	99	2629.2	2629.5 [M+H]^*+*^

^a^ RP-HPLC retention time

^b^ Percentage determined by HPLC at 220 nm from the crude reaction mixture

^c^ All peptides were analyzed by MALDI-TOF except for **BP134**, **BP216**, **BP236**, **tag54**, and **tag54-2** that were analyzed by ESI-MS

^d^ RP-HPLC analysis using conditions A (see Materials and Methods)

^e^ RP-HPLC analysis using conditions E (see Materials and Methods)

^f^ RP-HPLC analysis using conditions D (see Materials and Methods)

**Table 2:** Sequences, number of amino acids, retention times and purities on HPLC, and mass spectrometry data of peptides from the second set.

Peptide	Sequence	#Aa	*t*_R_ (min)^a^	Purity^b^ (%)	Theoretical monoisotopic *m/z*	Observed *m/z* ^c^
*BP134-melittin(10-19) or BP134-melittin(1-13)*						
**BP170**	KKLFKKILKYL-TTGLPALISW-OH	21	6.98^d^	90	2461.1	2461.4 [M+H]^*+*^
**BP171**	KKLFKKILKYL-AGPA-TTGLPALISW-OH	25	7.13^e^	87	2757.7	2757.8 [M+H]^*+*^
**BP207**	G-KKLFKKILKYL-AGPA-TTGLPALISW-OH	26	3.41^*f*^	85	2814.7	2814.6 [M+H]^*+*^
**BP208**	S-KKLFKKILKYL-AGPA-TTGLPALISW-OH	26	3.41^*f*^	85	2844.7	2844.5 [M+H]^*+*^
**BP172**	KKLFKKILKYL-TTGLPALISW-KDEL-OH	25	7.14^d^	87	2946.8	2946.8 [M+H]^*+*^
**BP173**	KKLFKKILKYL-AGPA-TTGLPALISW-KDEL-OH	29	7.04^d^	80	3242.9	3243.0 [M+H]^*+*^
**BP189**	KKLFKKILKYL-GIGAVLKVLTTGL-KDEL-OH	28	5.23^*g*^	98	3129.9	3130.0 [M+H]^*+*^
**BP217**	KKLFKKILKYL-TTGLPALIS-AGPA-SILAPLGTT-LYKLIKKFLKK-KDEL-OH	48	25.22^*h*^	99	5315.7 [M+H]^*+*^	1063.9 [M+5H]^5+^
*BP134-magainin(4-10)*						
**BP180**	KKLFKKILKYL-KFLHSAK-OH	18	6.56^d^	99	2233.4	2233.4 [M+H]^*+*^
**BP181**	KKLFKKILKYL-AGPA-KFLHSAK-OH	22	6.51^d^	99	2529.6	2529.6 [M+H]^*+*^
**BP211**	G-KKLFKKILKYL-AGPA-KFLHSAK-OH	23	3.15^*f*^	83	2586.6	2586.6 [M+H]^*+*^
**BP212**	S-KKLFKKILKYL-AGPA-KFLHSAK-OH	23	3.16^*f*^	82	2616.6	2616.6 [M+H]^*+*^
**BP182**	KKLFKKILKYL-KFLHSAK-KDEL-OH	22	6.13^d^	99	2718.4	2718.7 [M+H]^*+*^
**BP183**	KKLFKKILKYL-AGPA-KFLHSAK-KDEL-OH	26	6.11^d^	73	3014.7	3014.8 [M+H]^*+*^
**BP235**	KKLFKKILKYL-AGPA-KFLHSAK-AGPA-KDWEHLKDWEHLKDWEHL-KDEL-OH	48	17.32^*h*^	99	5738.8 [M+H]^*+*^	1148.5 [M+5H]^5+^
*BP134-magainin(1-10)*						
**BP176**	KKLFKKILKYL-GIGKFLHSAK-OH	21	6.48^d^	72	2460.1	2460.3 [M+H]^*+*^
**BP175**	KKLFKKILKYL-AGPA-GIGKFLHSAK-OH	25	6.52^e^	80	2756.7	2756.7 [M+H]^*+*^
**BP209**	G-KKLFKKILKYL-AGPA-GIGKFLHSAK-OH	26	3.29^*f*^	93	2813.7	2813.5 [M+H]^*+*^
**BP210**	S-KKLFKKILKYL-AGPA-GIGKFLHSAK-OH	26	3.30^*f*^	92	2843.7	2843.6 [M+H]^*+*^
**BP179**	KKLFKKILKYL-GIGKFLHSAK-KDEL-OH	25	6.44^d^	99	2945.7	2945.7 [M+H]^*+*^
**BP178**	KKLFKKILKYL-AGPA-GIGKFLHSAK-KDEL-OH	29	6.50^d^	90	3242.0	3242.7 [M+H]^*+*^
*BP134-cecropin A(25-37)*						
**BP188**	KKLFKKILKYL-AVAVVGQATQIAK-KDEL-OH	28	5.14^*g*^	99	3143.9	3144.0 [M+H]^*+*^
**BP215**	KKLFKKILKYL-AGPA-VAVVGQATQIAK-KDEL-OH	31	3.30^*f*^	92	3369.0	3369.0 [M+H]^*+*^
**BP190**	AVAVVGQATQIAK-KKLFKKILKYL-KDEL-OH	28	5.06^*g*^	98	3143.9	3144.0 [M+H]^*+*^

^a^ RP-HPLC retention time

^b^ Percentage determined by HPLC at 220 nm from the crude reaction mixture

^c^ All peptides were analyzed by MALDI-TOF except for peptides **BP217** and **BP235** that were analyzed by ESI-MS

^d^ RP-HPLC analysis using conditions A (see Materials and Methods)

^e^ RP-HPLC analysis using conditions B (see Materials and Methods)

^f^ RP-HPLC analysis using conditions E (see Materials and Methods)

^g^ RP-HPLC analysis using conditions C (see Materials and Methods)

^h^ RP-HPLC analysis using conditions D (see Materials and Methods)

The synthesis was performed following a standard Fmoc/*t*Bu solid-phase peptide synthesis methodology to yield C-terminal carboxylic acid sequences; overall purity was among 71-99% , except for three sequences that were obtained in 52-68% purity (Tables 1 and 2). Their molecular weights were confirmed by mass spectrometry.

### Antibacterial activity

 The peptides synthesized were tested for in vitro growth inhibition of *X. axonopodis* pv. vesicatoria, *P. syringae* pv. syringae and *E. amylovora* at 1.25, 2.5, 5.0, 7.5, 10, and 20 μM and compared to that of **BP134** (Tables 3 and 4).

**Table 3:** Antibacterial activity (MIC) against three plant pathogenic bacteria and hemolytic activity of peptides from the first set.

Peptide		MIC (μM)				Hemolysis^b^ (%)		
		*Xav^a^*	*Pss^a^*	*Ea^a^*		50 μM	150 μM	250 μM
*BP134 monomers*								
**BP134**		10-20	7.5-10	7.5-10		0	8	18
**BP199**		2.5-5.0	10-20	10-20		0	0	0
**BP214**		1.25-2.5	2.5-5.0	2.5-5.0		2	6	9
*BP134 dimers*								
**BP203**		1.25-2.5	5.0-7.5	10-20		91	93	95
**BP202**		<1.25	2.5-5.0	2.5-5.0		83	93	98
**BP193**		10-20	7.5-10	10-20		62	63	74
**BP195**		10-20	7.5-10	10-20		71	73	71
**BP200**		<1.25	7.5-10	>20		79	80	81
**BP198**		10-20	10-20	10-20		59	72	72
**BP192**		7.5-10	7.5-10	7.5-10		51	69	69
**BP213**		1.25-2.5	2.5-5.0	2.5-5.0		90	92	98
**BP194**		10-20	7.5-10	10-20		71	87	92
**BP196**		10-20	7.5-10	10-20		61	66	74
**BP236**		2.5-5.0	2.5-5.0	10-20		49	84	92
*BP134 trimers*								
**BP204**		2.5-5.0	10-20	>20		97	100	100
**BP201**		1.25-2.5	7.5-10	>20		74	81	83
**BP216**		10-20	>20	>20		89	98	100
*Tags*								
**tag54**		>100	>100	>100		0	0	1
**tag54-2**		>100	>100	>100		0	0	0

^a^ *Xav, Xanthomonas axonopodis* pv. vesicatoria; *Pss*, *Pseudomonas syringae* pv. syringae; *Ea*, *Erwinia amylovora*

^b^ Percent hemolysis plus confidence interval (α=0.05)

**Table 4:** Antibacterial activity (MIC) against three plant pathogenic bacteria and hemolytic activity of peptides from the second set.

Peptide		MIC (μM)				Hemolysis^b^ (%)		
		*Xav* ^a^	*Pss* ^a^	*Ea* ^a^		50 μM	150 μM	250 μM
*BP134-melittin(10-19) or BP134-melittin(1-13)*								
**BP170**		1.25-2.5	2.5-5.0	2.5-5.0		82	93	98
**BP171**		2.5-5.0	1.25-2.5	2.5-5.0		5	16	34
**BP207**		<1.25	1.25-2.5	1.25-2.5		5	36	58
**BP208**		<1.25	1.25-2.5	1.25-2.5		15	47	68
**BP172**		1.25-2.5	2.5-5.0	2.5-5.0		14	49	63
**BP173**		5.0-7.5	2.5-5.0	5.0-7.5		8	16	40
**BP189**		2.5-5.0	2.5-5.0	5.0-7.5		41	60	86
**BP217**		<1.25	2.5-5.0	2.5-5.0		91	100	100
*BP134-magainin*(4-10)								
**BP180**		2.5-5.0	2.5-5.0	2.5-5.0		12	54	58
**BP181**		2.5-5.0	1.25-2.5	2.5-5.0		0	0	0
**BP211**		<1.25	2.5-5.0	1.25-2.5		0	1	6
**BP212**		<1.25	2.5-5.0	2.5-5.0		0	5	12
**BP182**		1.25-2.5	1.25-2.5	2.5-5.0		1	38	59
**BP183**		5.0-7.5	7.5-10	2.5-5.0		1	4	5
**BP235**		2.5-5.0	2.5-5.0	2.5-5.0		0	0	0
*BP134-magainin(1-10)*								
**BP176**		2.5-5.0	2.5-5.0	5.0-7.5		3	44	59
**BP175**		1.25-2.5	2.5-5.0	5.0-7.5		6	14	32
**BP209**		<1.25	2.5-5.0	1.25-2.5		1	13	24
**BP210**		<1.25	2.5-5.0	1.25-2.5		0	17	30
**BP179**		2.5-5.0	2.5-5.0	5.0-7.5		0	12	35
**BP178**		2.5-5.0	2.5-5.0	2.5-5.0		0	3	25
*BP134-cecropin A(25-37)*								
**BP188**		2.5-5.0	1.25-2.5	2.5-5.0		10	24	42
**BP215**		1.25-2.5	2.5-5.0	2.5-5.0		0	3	14
**BP190**		5.0-7.5	>20	>20		11	17	32

^a^ *Xav, Xanthomonas axonopodis* pv. vesicatoria; *Pss*, *Pseudomonas syringae* pv. syringae; *Ea*, *Erwinia amylovora*

^b^ Percent hemolysis plus confidence interval (α=0.05)

From the analysis of the first set of peptides incorporating repeating units of **BP134**, we observed that the dimer **BP203** displayed higher activity than the trimer **BP204** against the three pathogens (Table 3). Moreover, **BP203** was more active than the monomer **BP134** against *X. axonopodis* pv. vesicatoria (1.25-2.5 μM vs 10-20 μM) and *P. syringae* pv. syringae (5.0-7.5 μM vs 7.5-10 μM). The introduction of an AGPA moiety as a hinge between two **BP134** units afforded peptides displaying higher activity (compare **BP203** and **BP202**; **BP198** and **BP192**; **BP204** and **BP201**). In contrast, the incorporation of an AGPA moiety at the C-terminus of **BP134** or of **BP202** led to peptides **BP199** and **BP200**, respectively, with enhanced or similar activity against *X. axonopodis* pv. vesicatoria (2.5-5.0 μM and <1.25 μM), and with decreased activity against the other two pathogens. When a KDEL moiety was introduced at the C-terminus of **BP134**, an increase of the activity was observed against the three bacteria (**BP214**, 1.25-5.0 μM). However, the presence of a KDEL unit at the C-terminus of a dimer sequence maintained or decreased the activity, as shown for **BP203** vs **BP198**, **BP202** vs **BP192**, **BP193** vs **BP194**, and **BP195** vs **BP196**. The same behaviour was observed for the trimer peptides **BP201** and **BP216**. The incorporation of a Gly or a Ser residue at the N-terminus of **BP192** (5.0-10 μM) and **BP202** (<1.25-5.0 μM) rendered peptides **BP193-BP196** with higher MIC values (7.5-20 μM). The epitope tag peptides **tag54** and **tag54-2**, the latter incorporating a KDEL unit at the C-terminus, were not active against the three bacteria. However, the introduction of **tag54-2** into **BP200** resulted in peptide **BP236** with increased activity against *P. syringae* pv. syringae (2.5-5.0 μM) and *E. amylovora* (10-20 μM), and with reduced activity against *X. axonopodis* pv. vesicatoria (2.5-5.0 μM). Peptide dimer **BP213**, bearing a reversed peptide sequence from **BP134** at the C-terminus, was slightly more active than **BP192** against the three pathogens (1.25-5.0 μM vs 5.0-10 μM). From this set of peptides, the most active sequences were **BP202**, **BP213** and **BP214**.

Concerning the second set of peptides, incorporating a combination of one unit of **BP134** with fragments of the natural antimicrobial peptides melittin, magainin and cecropin A, we observed that peptide dimers **BP170**, **BP176** and **BP180** displayed lower MIC values (1.25-7.5 μM) than **BP134** (Table 4). In general, the incorporation of an AGPA moiety into the sequence of peptides **BP170**, **BP172**, **BP176**, **BP179**, **BP180**, **BP182**, and **BP188** did not significantly influence the activity rendering peptides **BP171**, **BP173**, **BP175**, **BP178**, **BP181**, **BP183** and **BP215**, respectively, with MIC values of 1.25-10 μM. Sequences **BP172**, **BP173**, **BP178**, **BP179**, **BP182** and **BP183**, bearing a KDEL moiety at the C-terminus, were as active or slightly less active (1.25-10 μM) than the corresponding peptides **BP170**, **BP171**, **BP175**, **BP176**, **BP180** and **BP181** (1.25-7.5 μM). In general, the derivatization of **BP171**, **BP175** and **BP181** at the N-terminus with a Gly or a Ser residue resulted in peptides **BP207**-**BP212** with slightly lower MIC values (<1.25-5.0 μM). Peptide **BP235**, derived from **BP134** and magainin(4-10) and bearing two AGPA moieties, **tag54-2**, and KDEL, showed similar activity than **BP181** (2.5-5.0 μM). Peptide **BP217**, which resulted from the combination of **BP170** and a reversed peptide sequence from **BP170** linked with an AGPA moiety and bearing KDEL at the C-terminus, displayed similar activity than **BP170** (<1.25-5.0 μM). The analogue **BP172**, resulting from the combination of **BP134** and melittin(10-19), was more active (1.25-5.0 μM) than **BP189** bearing a melittin(1-13) fragment. When the **BP134** and cecropin A(25-37) fragments in **BP188** (1.25-5.0 μM) were inverted, the resulting peptide **BP190** was poorly active (5.0 - >20 μM). The best peptides of this second set were **BP207**, **BP208**, **BP209**, **BP210**, and **BP211** which also displayed higher activity than the most active peptides from the first set.

### Hemolytic activity

 The toxicity of peptides to eukaryotic cells was determined as the ability to lyse erythrocytes in comparison to melittin. Percent hemolysis at 50, 150 and 250 μM is shown in Tables 3 and 4.

Among the peptides of the first set, the two **BP134** monomeric analogues **BP199** and **BP214** were not hemolytic even at 250 μM (0-9%), whereas the dimer **BP203** and the trimer **BP204** were highly hemolytic (95-100%) (Table 3). Peptides incorporating an AGPA moiety either as a hinge or at the C-terminus (**BP192** and **BP199-BP202**) exhibited similar hemolytic activity than the corresponding peptides that do not incorporate this moiety (**BP134**, **BP198**, **BP203**, and **BP204**). The incorporation of a KDEL moiety at the C-terminus of **BP134**, **BP193**, **BP195**, **BP201**, **BP202**, and **BP203** resulted in peptides **BP214**, **BP194**, **BP196**, **BP216**, **BP192**, and **BP198**, respectively, that did not display a clear hemolytic activity pattern. The modification of the N-terminus of peptide **BP202** with a Gly or a Ser residue led to peptides **BP193** and **BP195** with lower hemolysis (71-74% at 250 μM). In contrast, when the same derivatization was performed on **BP192**, the resulting peptides **BP194** and **BP196** were slightly more hemolytic (74-92% at 250 μM). The presence of the epitope tag **tag54-2** (not hemolytic) at the C-terminus of **BP236** did not significantly influence the hemolytic activity as compared to **BP200** (92% vs 81% at 250 μM). Peptide dimer **BP213**, bearing a reversed peptide sequence from **BP134** at the C-terminus, was more hemolytic (98% at 250 μM) than its analogue **BP192** (69% at 250 μM). Peptide monomers **BP199** and **BP214** were the least hemolytic sequences from this set (0-9% at 250 μM) and **BP192**, **BP193**, **BP195**, **BP196** and **BP198** displayed <75% hemolysis at 250 μM.

Regarding the second set of peptides (Table 4), the combination of one unit of **BP134** with melittin(10-19), magainin(4-10) and magainin(1-10) resulted in analogues **BP170**, **BP180** and **BP176**, respectively, with a significantly higher hemolysis percentage (58-98% at 250 μM) than that of **BP134** (18% at 250 μM). Among them, the most hemolytic was **BP170**. Peptides containing an AGPA moiety as a hinge, **BP171**, **BP173**, **BP175**, **BP178**, **BP181**, **BP183** and **BP215**, were less hemolytic (0-40% at 250 μM) than the corresponding analogues **BP170**, **BP172**, **BP176**, **BP179**, **BP180**, **BP182**, and **BP188** (35-98% at 250 μM). The incorporation of a KDEL moiety at the C-terminus of **BP170**, **BP171**, **BP175**, **BP176**, **BP180** and **BP181** rendered peptides **BP172**, **BP173**, **BP178**, **BP179**, **BP182** and **BP183** with similar or lower hemolysis. When peptides **BP171**, **BP175** and **BP181** were modified at the N-terminus with a Gly or a Ser residue, the resulting sequences **BP207**-**BP212** showed a comparable hemolytic percentage being those incorporating a Ser residue the most hemolytic. **BP181** and its analogue **BP235**, incorporating a **BP134** unit and a magainin(4-10) fragment together with two AGPA moieties and **tag54-2**, were not hemolytic even at 250 μM. Similarly to **BP170**, its analogues **BP189** and **BP217** were highly hemolytic. The derivatives containing a **BP134** unit and a cecropin A(25-37) fragment, **BP188** and **BP190**, displayed similar hemolysis (42% and 32% at 250 μM, respectively). The least hemolytic peptides from this second set are **BP178**, **BP181**, **BP183**, **BP209**, **BP211**, **BP212**, **BP215**, and **BP235** which displayed hemolysis ≤25% at 250 μM.

### Phytotoxicity

**Figure 1:**
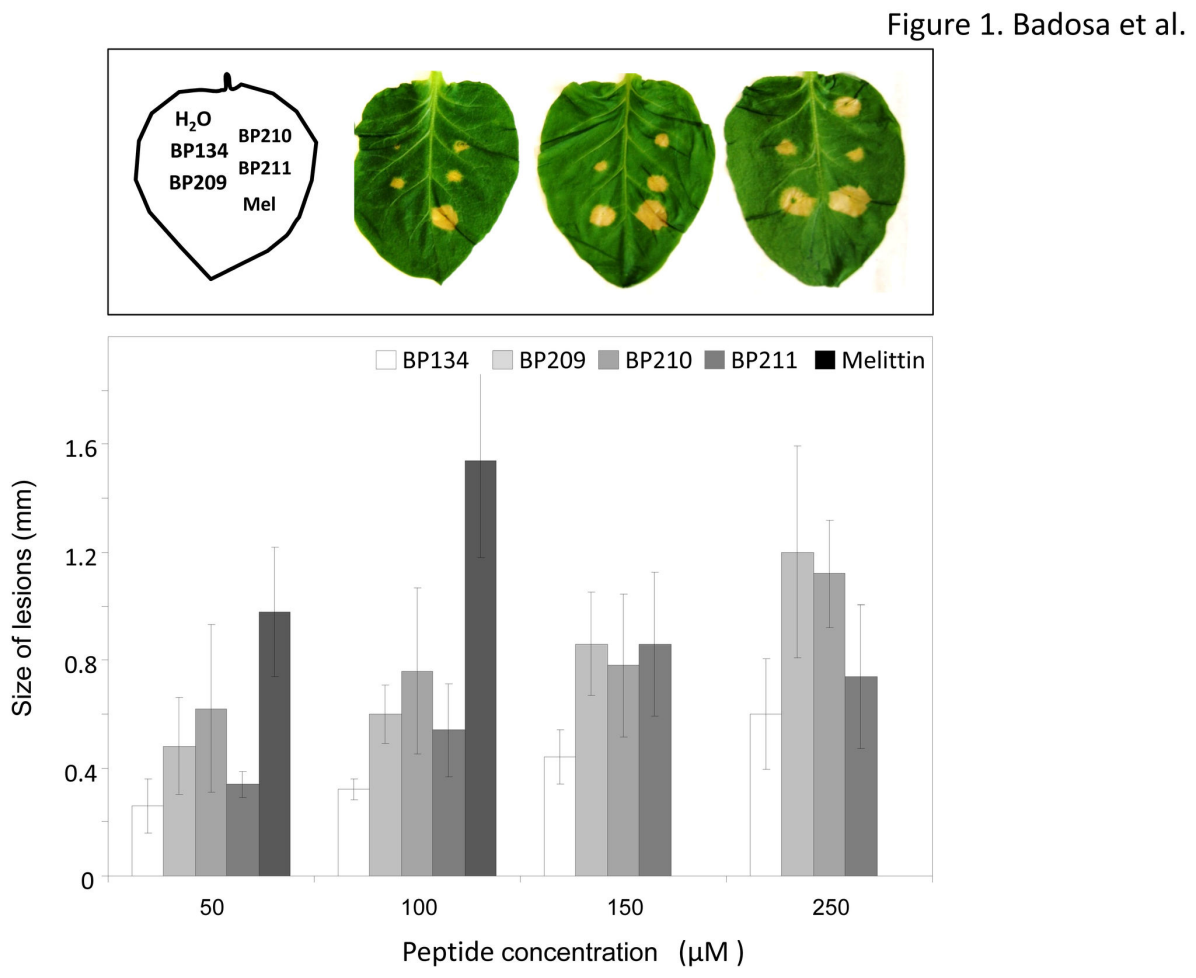
Phytotoxicity of selected antimicrobial peptides BP209, BP210 and BP211, in comparison to BP134 and melittin. Peptides were infiltrated in tobacco plant leaves at different concentrations (50, 100, 150 and 250 μM). The size of lesions is considered as a measure of phytotoxicity. Peptide solutions at given concentrations were micro-infiltrated into the mesophyll of leaves in plants and incubated for three days. Vertical bars within each column indicate confidence interval of the mean.

**Figure 2:**
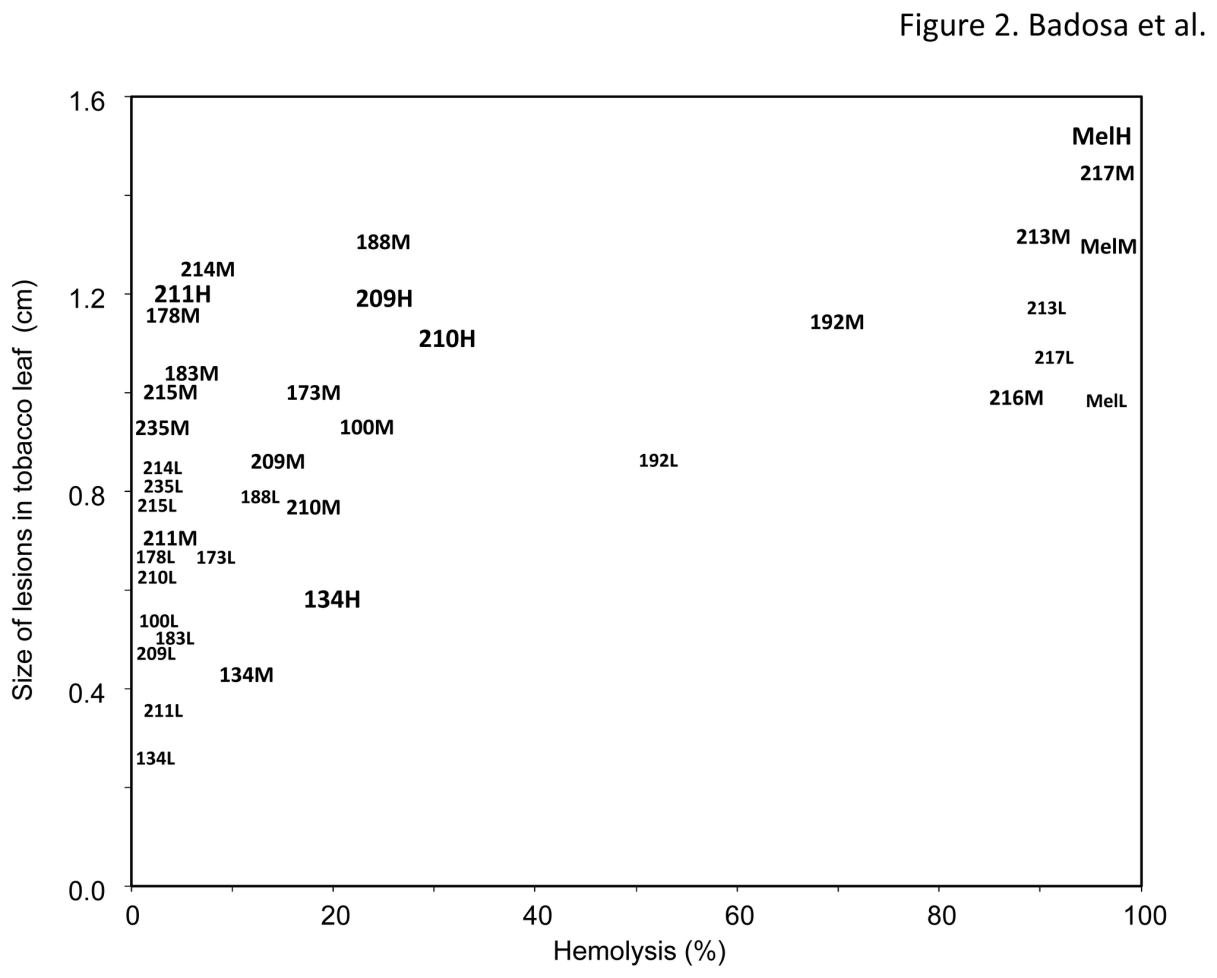
Relationship between the phytotoxicity of antimicrobial peptides and their hemolytic activity. The assay was performed at 50 (low, L), 150 (medium, M) and 250 μM (high, H). For some peptides not all concentrations were assayed.

 All **BP134** derived peptides were more phytotoxic than the original monomer (Figures 1 and 2). However, phytotoxicity was only observed at concentrations 10-to-50 times higher than the MIC, as in the case of hemolytic activity. Tobacco leaves responded in a very quick and selective manner to peptides, and a clear dose-response effect was observed for some of the best antibacterial peptides (**BP134**, **BP209**, **BP210**, and **BP211**), with practically a linear increase from 50 to 250 μM (Figure 1). Melittin was the most phytotoxic peptide, inducing lesions of around 2 cm diameter. The most phytotoxic **BP134** derivatives were **BP214** (BP134-KDEL), **BP192** and **BP213** (BP134 dimer derivatives), **BP217** (BP134-melittin(10-19) dimer derivative), **BP178** (BP134-AGPA-magainin(1-10)-KDEL), and **BP188** (BP134-cecropin A(25-37)-KDEL) (Figure 2). Peptides with moderate phytotoxicity were **BP100**, **BP173** (BP134-AGPA-melittin(10-19)-KDEL), **BP183**, **BP211**, and **BP235** (BP134-AGPA-magainin(4-10) derivatives), **BP209** and **BP210** (BP134-AGPA-magainin(1-10) derivatives), **BP215** (BP134-AGPA-cecropin A(25-37)-KDEL), and **BP216** (BP134 trimer derivative). 

### Relationship between phytotoxicity and hemolytic activity

 When phytoxicity was plot against hemolytic activity (Figure 2) two different patterns were observed. Peptides **BP192**, **BP213**, **BP216** and **BP217** were strongly phytotoxic and hemolytic, with similar values as melittin at the same concentrations. In contrast, peptides **BP173**, **BP178**, **BP183**, **BP188**, **BP209**, **BP210, BP211, BP214, BP215**, and **BP235** were low or very low hemolytic but they unexpectedly caused moderate to significant lesions in tobacco leaves upon infiltration. 

## Discussion

 The undecapeptide **BP100** (KKLFKKILKYL-NH_2_), identified from a library of cecropin A-melittin hybrid peptides, has significant activity against *X. axonopodis* pv. vesicatoria, *P. syringae* pv. syringae and *E. amylovora* (MIC of 2.5-7.5 μM) and low hemolysis (22% at 150 μM) [17]. Since it is known that peptide expression by plants requires a minimum chain length and that these peptides are produced as C-terminal carboxylic acids, here we describe **BP100** analogues containing from 15 to 52 residues and a carboxylic acid group at the C-terminus. The C-terminal carboxylic acid undecapeptide **BP134** (KKLFKKILKYL-OH) was also included for comparison purposes. In designing **BP134** analogues, we incorporated repeated units of **BP134** or a combination of one **BP134** sequence with a fragment of a natural antimicrobial peptide, in particular, melittin(10-19), melittin(1-13), magainin(4-10), magainin(1-10) or cecropin A(25-37). Moreover, we modified these peptides by introducing one or two AGPA hinges as stabilization/distortion moiety, a KDEL fragment at the C-terminus as a signal for permanent retention of peptides in the endoplasmic reticulum, a Gly or a Ser residue at the N-terminus as a TEV protease recognition site and the epitope tag KDWEHLKDWEHLKDWEHL (**tag54**) at the C-terminus for peptide detection and purification. We studied the influence of these modifications on the antimicrobial and hemolytic activities.

The combination of **BP134** with another unit of this peptide or with a fragment of melittin or magainin led to sequences with higher antibacterial activity, being the hybrid BP134-melittin(10-19) (BP170) the most active. Moreover, the elongation of a peptide sequence with an AGPA hinge, a KDEL unit or the tag epitope **tag54-2** do not significantly influence peptide activity. Notably, the modification of the N-terminus in peptides **BP171** (BP134-AGPA-melittin(10-19)), **BP175** (BP134-AGPA-magainin(1-10)) and **BP181** (BP134-AGPA-magainin(4-10)) with a Gly or a Ser residue led to an increase of the antibacterial activity. Peptides with this Gly or Ser residue are generated upon hydrolysis with the TEV protease over peptide-protein fusions in certain strategies for heterologous expression in plants [38]. The resulting sequences **BP207**-**BP212** displayed similar activities and were among the best peptides identified in this study (MIC of <1.25 to 5.0 μM). These results also show the effect of small sequence modifications on the biological activity of these peptides [17,42-44] and are in agreement with previous studies reporting that the length and sequence are among the most important factors for biological activity of antimicrobial peptides [45].

The order of the peptide fragments is crucial for the antibacterial activity. For example, the hybrid **BP188**, containing the cationic peptide **BP134** at the N-terminus and the hydrophobic cecropin A(25-37) fragment at the C-terminus is highly active against the three pathogens, whereas the analogue **BP190** containing cecropin A(25-37)-BP134 is only active against *X. axonopodis* pv. vesicatoria and with higher MIC values than **BP188**. This result is in agreement with the structural features of cecropins necessary for antibacterial activity which include a basic N-terminus and a hydrophobic C-terminus [46]. 

Interestingly, the peptides incorporating a combination of a normal and a reversed peptide sequence, like **BP213** and **BP217**, showed higher or similar activity than the normal peptides **BP192** and **BP170**, respectively. A similar behavior has been previously reported for reversed peptides derived from the antimicrobial peptides cecropin, melittin or magainin [47-51].

Peptide toxicity has been evaluated against red blood human cells thanks to the highly standardized methods available and because data can be compared straightforwardly with other reports, mainly dealing with human pathogens. In general, the elongation of the **BP134** sequence with another **BP134** unit or with a fragment of a natural antimicrobial peptide, produced an increase of the hemolytic activity. The most hemolytic peptides were those incorporating two **BP134** units and those derived from melittin(10-19) and melittin(1-13), while the derivatives obtained from magainin and cecropin A fragments displayed the lowest hemolysis. Among the latter, six peptides were less hemolytic than **BP134** (0-14% at 250 μM). The hemolysis observed for the analogues designed from melittin, magainin and cecropin A correlated with that of the natural peptides. In fact, melittin has been described to be very cytotoxic for erythrocytes, whereas magainin and cecropin A have no toxicity [52,53]. Surprisingly, even though **BP134** is low hemolytic, the dimer **BP203** and its analogues showed high hemolysis.

The incorporation of an AGPA hinge to peptides containing two units of **BP134** did not influence the hemolytic activity. Notably, when this hinge was introduced in peptides including a melittin, magainin or cecropin A fragment, the cytotoxicity decreased significantly. The other modifications such as the derivatization with a Gly or a Ser residue at the N-terminus or with **tag54-2** at the C-terminus resulted in peptides with comparable hemolysis. Peptides bearing a Gly residue were slightly less hemolytic than those containing a Ser. On the other hand, the elongation of a peptide with a reversed sequence afforded compounds highly hemolytic (**BP213** and **BP217**). Interestingly, the most active peptides **BP207**-**BP211** showed low hemolysis (6-68% at 250 μM), being **BP209**, **BP210** (BP134-magainin(1-10) derivatives) and **BP211** (BP134-magainin(4-10) derivative) the sequences with the best balance between antibacterial and hemolytic activities. 

In relation to phytotoxicity, all peptides were more phytotoxic than **BP134** and a dose-response direct relationship was observed between the concentration of peptide and the development of lesion in tobacco. However, best peptides in terms of high antibacterial and low hemolytic activities (**BP209**, **BP210** and **BP211**) showed a moderate phytotoxicity. In agreement with our observation is the report that the constitutive expression of transgenes encoding certain **BP134** analogues (**BP192**, **BP213**, **BP216**, **BP217**) has a negative impact on rice plant regeneration upon callus transformation [41]. 

Interestingly, several peptides incorporating a **BP134** unit and melittin, magainin or cecropin A fragment, showed low hemolytic activity, but moderate or high phytotoxicity. It could be possible that these peptides have no targets into the erythrocyte membrane, but affect tobacco plant cell membranes. However, a second possibility is that these peptides are elicitors of the hypersensitivity reaction in tobacco leaves, but this cannot be distinguished from phytotoxicity based on lesion symptoms alone as used here, and would require additional analysis. The possibility of having peptides in the CECMEL11 library with defense elicitation properties is in agreement with other studies reporting elicitation of defense responses by peptides in BY2 tobacco cells and protoplasts [54] and in cucumber and *Arabidopsis* leaves [55]. 

In the present work, phytotoxicity has been assessed by leaf infiltration into tobacco as a model and it may differ in other plant systems that can be used to express **BP134** derivatives. However, a significant correlation has been reported between toxicity in tobacco leaves and rice seed germination for 10 relevant peptides (**BP134**, **BP235**, **BP183**, **BP173**, **BP178**, **BP215**, **BP217**, **BP213**, and **BP192**) which have been included in the present report [56].

In summary, we have described a convenient strategy for the development of peptides to be expressed by plants with high antibacterial activity, low hemolysis and moderate phytotoxicity. The structural features that confer these biological properties are the presence of an AGPA hinge together with a Gly residue at the N-terminus as a protease recognition site. Moreover, since the presence of a KDEL unit or **tag54-2** at the C-terminus of a peptide sequence do not influence significantly in its biological activity, these moieties can be introduced enabling the design of compounds that can be retained in the endoplasmic reticulum and recognized by a complementary epitope. Interestingly, the best peptides in terms of high antibacterial and low hemolytic activities, with moderate phytotoxicity were **BP209**, **BP210** and **BP211**. Current research by our laboratory involves the expression of several of these peptides in rice plants using diverse strategies to direct expression either to the whole plant or to seed endosperm or embryo, that will permit to verify the main conclusions of the present study. 
